# Data from SymSPAN and OSPAN working memory capacity tasks in online and laboratory settings

**DOI:** 10.1016/j.dib.2025.111923

**Published:** 2025-07-25

**Authors:** Michał Wereszczyński, Paulina Chwiłka, Ewa Smołka, Ewa Ilczuk, Sezin Öner, Krystian Barzykowski

**Affiliations:** a Applied Memory Research Laboratory, Institute of Psychology, Faculty of Philosophy, Jagiellonian University, Krakow, Poland; b Department of Psychology, Kadir Has University, Turkey

**Keywords:** Working memory capacity, Operation span task, Symmetry span task, Online experiment

## Abstract

The present dataset comprises the performance of adult participants on two experimental tasks designed to measure working memory capacity: the Symmetry Span (SymSPAN) and Operation Span (OSPAN) tasks. Initially, a large sample of 566 participants completed these tasks online. From this pool, a random subset of individuals representing low, medium, and high levels of working memory capacity were invited to participate in two laboratory sessions. In these sessions, spaced one week apart, participants completed the same tasks again. The dataset includes complete performance data from both tasks, along with demographic information such as participants’ age and gender. This relatively large dataset offers valuable opportunities for exploratory research on working memory capacity, including analyses of its relative stability, variations over time and across testing environments, individual differences, and contributions to meta-analyses.

Specifications Table

**Table:** 

Subject	Psychology: Experimental and cognitive psychology
Specific subject area	*Cognitive Psychology; Working Memory Capacity, Symmetry Span task, Operation Span Task; Online Behavioural Experiment*
Type of data	Excel.xls
Data collection	The dataset includes performance measures from adult participants on two experimental tasks assessing working memory: the Operation Span Task (OSPAN) and the Symmetry Span Task (SymSPAN). Each task was completed by participants on separate days in both an online (web-based) setting and two separate offline (laboratory-based) sessions. All tasks were administered using Inquisit Web software 7.1. Data collection for the online phase took place between May 5, 2023, and December 6, 2023, while the laboratory phase was conducted between February 23, 2024, and June 3, 2024. Initially, a large pool of participants was invited to complete the working memory tasks online, resulting in a final sample of 566 participants. Recruitment for the online phase was carried out via social media (Facebook), university advertisements, and flyers. Based on participants’ performance in the online tasks, a subset of 207 individuals representing a range of working memory capacities was randomly selected and invited to participate in the laboratory-based sessions.
Data source location	Applied Memory Research Laboratory, Institute of Psychology, Faculty of Philosophy, Jagiellonian University, Krakow, Poland
Data accessibility	All data are attached to the paper. Repository name: RODBUK Data identification number: 10.57903/UJ/JXOPW3 Direct URL to data: https://doi.org/10.57903/UJ/JXOPW3
Related research article	None

## 1 Value of the Data

• This design allows researchers to compare performance in working memory capacity tasks across online and offline conditions, making the dataset particularly valuable for studies investigating the effects of testing environments on working memory assessment.
• The data may contribute to further development of future web-based behavioural experiments and facilitate exploratory research on how the testing context influences cognitive task performance.
• Furthermore, the substantial sample sizes of both the online and the laboratory sessions offer a useful resource for researchers aiming to establish normative data or conduct meta-analyses on working memory performance using standardized tasks.
• This dataset offers a comprehensive range of working memory performance measures that enable both between-setting comparisons (e.g., laboratory vs. web-based environments) and between-session comparisons (e.g., first vs. second laboratory session). These features make the data suitable for in-depth analyses of performance variability in classic experimental working memory tasks.

## 2 Background

The dataset presented in this article reflects the working memory capacity task performance of adult participants across three experimental sessions: one conducted online and two held in a laboratory setting. The data were collected as a part of a larger research project aimed at investigating the relationship between working memory capacity (WMC) and the occurrence of involuntary autobiographical memories (IAMs) as well as involuntary future-oriented thoughts (IFTs) [1]. IAMs refer to spontaneous recollections of personally experienced past events (e.g., *recalling playing a favourite childhood game*), whereas IFTs involve thoughts about possible future events (e.g., *planning an upcoming vacation*). Although both IAMs and IFTs typically arise spontaneously and with minimal cognitive effort [2], a consistent finding in the literature is that their frequency diminishes under conditions of high cognitive demand [[2], [3], [4], [5], [6], [7], [8]]. This phenomenon, referred to as cognitive load dependency, may be modulated by individual differences in working memory capacity. To investigate these processes, participants were initially recruited to complete the Symmetry Span and Operation Span tasks online. The results of these tasks served to classify participants into low, medium, or high WMC groups. Participants randomly selected from these three groups were invited to the laboratory sessions. Each laboratory session started with a task measuring the frequency and characteristics of IAMs and IFTs under either low or high cognitive load conditions (operationalised as an easy or difficult n-back task). After that, participants completed the SymSPAN and OSPAN tasks. This paper focuses exclusively on participants’ performance in the WMC tasks; data concerning the frequency and content of IAMs and IFTs will be reported in a separate publication [1].

## 3 Data Description

The present dataset includes several files. The main file, Data_Base_WMC_Lab-Online.xlsx, contains summary performance data for participants on two working memory tasks: the Operation Span Task (OSPAN) and the Symmetry Span Task (SymSPAN). These data were extracted from files generated by the Inquisit Web platform. Table 1 within the file provides descriptions of the variables related to participants’ performance on both tasks. In addition to the main dataset, raw data for each task and session are available in the following Excel files: ONLINE_OSPAN_raw.xlsx,

**Table 1:** Study online and laboratory: descriptions of variables. Please note that the variable descriptions were taken directly from the respective user manuals for the symmetry span task (SymSPAN manual) and the operation span task (OSPAN manual).

Variable	Description
ID	Participant’s individual ID number
N-back condition	Condition of the n-back task the participant performed before the SymSPAN or OSPAN session.
gender	Gender of the participant (possible answers: male, female, other (*inna* in the file), prefer not to answer)
age	Age of the participant
startdate	The day the session was completed
starttime	The time the session started
elapsedtime	Time taken to complete the session
sspan (absolute score)	Tracks the sum of all correctly recalled squares of correctly recalled sets during the SymSPAN session. (Max for the default experimental set-up: 42).
totalcorrectsquares	The sum of all correctly recalled squares during the SymSPAN session
totalrecalledsets	The number of all correctly recalled sets during the SymSPAN session
symmetrytotalerrors	Tracks the total number of symmetry errors (both speed and accuracy problems) during the SymSPAN session
symmetryspeederrors	Tracks symmetry problem errors due to speed (i.e., was too slow to make a decision) during the SymSPAN session
symmetryaccerrors	Tracks symmetry problem errors due to accuracy (i.e., selected the incorrect answer) during the SymSPAN session
symmetryaccuracy	Percent correct symmetry problems during the SymSPAN session
ospan (absolute score)	The OSPAN score uses the traditional “absolute ospan” scoring method. It is the sum of all perfectly recalled sets. So, for example, if an individual correctly recalled 2 letters in a set size of 2, 3 letters in a set size of 3, and 3 letters in a set size of 4, their OSPAN score would be 5 (2 + 3 + 0).
totalcorrectletters	The total number of letters recalled in the correct position during the OSPAN session
mathtotalerrors	The number of all errors (speed or accuracy) made during math problems within the current block (during the OSPAN session)
mathspeederrors	The number of times the participant ran out of time when solving the math problems within the current block during the OSPAN session
math accuracy	Percentage of correctly solved math problems within the current block during the OSPAN session
mathaccerrors	The number of incorrectly solved math problems within the current block during the OSPAN session
testing context: *At the beginning of the study, we asked you to follow certain guidelines to maximize the reliability of your participation. By “reliably,” we mean completing the tasks in a way that reflects your typical functioning, without being affected by unfavorable environmental conditions (*e.g.*, noise) or physical states (*e.g.*, severe fatigue).* *We would now like to know whether you were able to meet these guidelines.* *Please answer honestly and truthfully, as this information will help us determine how best to analyze your results in this study.* *The conditions under which you performed the online tasks (*e.g.*, volume of sounds, presence of other people, level of fatigue and attention, etc.*)	Assessment of the conditions (e.g., noise intensity, presence of other people, level of fatigue and attention, etc.) in which the participant performed the task (favourable or neutral for reliable performance of the task)
well-being after N-back (*Please indicate what is your mood at this moment:*)	Assessment of well-being after performing the N-back task (on a 1–7-point scale): *1 = Very bad* *2 = Bad* *3 = Rather bad* *4 = Neutral* *5 = Rather good* *6 = Good* *7 = Very good*
physical tiredness after N-back (*How physically tired are you at this moment?*)	Assessment of physical tiredness after performing the N-back task (on a 1–7-point scale): *1 = I am not physically tired at all* *2 = I am not physically tired* *3 = Rather I am not physically tired* *4 = I am somewhat physically tired* *5 = Rather I am physically tired* *6 = I am physically tired* *7 = I am very physically tired*
mental fatigue after N-back (*How mentally tired are you at this moment?)*	Assessment of mental fatigue after performing the N-back task (on a 1–7-point scale): *1 = I am not mentally tired at all* *2 = I am not mentally tired* *3 = Rather I am not mentally tired* *4 = I am somewhat mentally tired* *5 = Rather I am mentally tired* *6 = I am mentally tired* *7 = I am very mentally tired*
task evaluation after N-back (*To what extent do you think the task performance was tiring?)*	Participant’s subjective assessment of their performance during the N-back task (on a 1–7-point scale): *1 = It was not tiring at all* *2 = It was not tiring* *3 = Rather It was not tiring at all* *4 = Difficult to say* *5 = Rather It was tiring* *6 = It was tiring* *7 = It was very tiring*
N-back elapsedtime	Time taken to complete the N-back task.

All variables (except ID online, ID LAB, gender and age) occur in each condition. Prefixes before each variable in the dataset describe the condition from which the variable comes. First, we describe whether the session was conducted online or in the first or second session in the laboratory (ONLINE_x, LAB1_x, LAB2_x). Then, we indicate which procedure was performed (x_OSPAN_x, x_SYMSPAN_x, x_SURVEY_x). For example, the start date of the Symmetry Span administered during the second laboratory session is named LAB2_SYMSPAN_startdate.

ONLINE_OSPAN_summary.xlsx,

ONLINE_SYMSPAN_raw.xlsx,

ONLINE_SYMSPAN_summary.xlsx,

L1_OSPAN_raw.xlsx,

L1_OSPAN_summary.xlsx,

L1_SYMSPAN_raw.xlsx,

L1_SYMSPAN_summary.xlsx,

L2_OSPAN_raw.xlsx,

L2_OSPAN_summary.xlsx,

L2_SYMSPAN_raw.xlsx,

L2_SYMSPAN_summary.xlsx.

In these filenames, “online” refers to data collected during the web-based session; “L1” and “L2” refer to the first and second laboratory-based sessions, respectively.

## 4 Experimental Design, Materials and Methods

### 4.1 Participants

The full sample consisted of 566 participants, including 406 women, 138 men, 13 individuals who identified as “other”, and 9 who preferred not to disclose their gender. Participants’ ages ranged from 18 to 40 years (*M*=22.01, SD = 3.72). The recruitment for this initial online phase was carried out via social media (Facebook), university advertisements, and flyers. Based on participants’ performance in the online tasks, a subsample of 207 adults took part in the two laboratory sessions. Importantly, of the 226 participants who were invited to the laboratory, 19 did not complete the working memory tasks in at least one laboratory session, resulting in an attrition rate of approximately 8.41% between the first and second laboratory sessions. The final group of 207 adults included 146 women, 55 men, 4 individuals who identified as “other”, and 2 who preferred not to disclose their gender, with ages ranging from 18 to 40 years (*M*= 21.72, SD = 3.37). Participants received a modest reward of 100 PLN (approximately 25 USD) for their participation.

In order to ensure variability in individual differences in working memory capacity, the participants were split into three groups (low, medium, and high) on the basis of their composite working memory scores (for a similar approach in terms of the composite inhibitory control capacity score, see also [9]). These scores were calculated as the mean of Z-transformed results of two computerized complex span tasks (OSPAN and SymSPAN). Participants were then sorted by their composite score and divided into three approximately equal-sized groups corresponding to the lowest, middle, and highest third of the distribution. Participants from each group were randomly selected and invited to participate in the laboratory phase.

### 4.2 Procedure and materials

We used two well-known tasks to measure working memory capacity [10]: the Automated Operation Span Task and the Symmetry Span task. We utilised Inquisit Web software (Millisecond software) [11]. Importantly, we used identical versions of these tasks in both the online and laboratory sessions.

**Operation Span Task (OSPAN):** In this task, participants are required to mentally solve simple arithmetic problems (e.g., (2 ×3) + 2 = ?) and then determine whether a number presented on the screen matches the solution they have just calculated. Following this, a letter (e.g., B, Z) briefly appears on the screen. After a series of 3 to 7 such trials, participants are asked to recall the letters in the exact order they were presented. Working memory capacity is indexed by the absolute OSPAN score, which reflects the total number of letters correctly recalled in the correct serial order (for scoring details see also [20]). More precisely, the absolute OSPAN (and likewise the SymSPAN) score is calculated as the sum of all correctly recalled sets—that is, those in which the entire letter sequence was reproduced in the correct order. For example, if a participant correctly recalls 2 letters in a set of size 2, 3 in a set of 3, 4 in a set of 4, but only 3 in a set of 5 (thus not fully correct), their absolute OSPAN score would be 9 (2 + 3 + 4 + 0). In contrast, the total number of correct letters—regardless of whether the full set was correct—is calculated as the sum of all letters recalled in the correct position, which in this example would be 12 (2 + 3 + 4 + 3). Higher scores indicate higher working memory capacity. The entire task takes approximately 20 min and includes 75 letters and 75 math problems across three sets, each consisting of trials, with set sizes ranging from 3 to 7 letters. A structured practice phase precedes the main task, comprising (a) four trials involving recall of letter sequences (set sizes 2 and 3), (b) fifteen practice trials of the math task alone, and (c) three combined practice trials involving two-letter recall sequences interspersed with arithmetic problems. To minimize the possibility of participants rehearsing the letter sequences while solving the math problems, the program records each participant’s average reaction time (RT) during the 15 math practice trials and calculates a personalized cut-off time (*M* + 2.5 SD) for the main task [12]. If participants exceed this cut-off, the verification screen is skipped, and the trial is recorded as incorrect. This same timing mechanism is also applied in the Symmetry Span task.

**Symmetry Span Task (SymSPAN):** The Symmetry Span Task, adapted from [13], is designed to assess spatial working memory capacity. In this task, participants are required to recall sequences of red-square locations (ranging from 2 to 5 items) within a 4 × 4 matrix, while simultaneously performing a symmetry judgment task. Each trial begins with the presentation of an 8 × 8 black-and-white matrix pattern; participants must decide whether the pattern is vertically symmetrical (approximately half of the patterns are). Immediately after each symmetry judgment, a red square briefly appears in one of the cells of a 4 × 4 matrix. This alternating sequence of symmetry judgments and red-square presentations continues for the length of the trial (i.e., set size). At the end of each trial, participants are presented with a blank 4 × 4 matrix and are instructed to recall the locations of the red squares in the order in which they appeared by clicking on the appropriate cells. As with the OSPAN task, spatial working memory performance is indexed by the absolute SymSPAN score, calculated as the total number of correctly recalled sets, i.e., trials in which all red-square locations were recalled in the correct serial order. For example, if a participant correctly recalls all red-square positions in three trials of set sizes 2, 3, and 4, but only recalls 3 out of 5 locations correctly in a set size of 5, their absolute SymSPAN score would be 9 (2 + 3 + 4 + 0). In contrast, their total number of correct locations would be 12 (2 + 3 + 4 + 3). Higher scores indicate higher spatial working memory capacity. The SymSPAN task takes approximately 15–20 min and includes a total of 12 trials with varying set sizes. Prior to the main task, participants complete a structured practice session composed of (a) four trials involving only the red-square location recall task (set sizes 2 and 3), (b) fifteen symmetry-judgment practice trials, and (c) three combined practice trials in which a set of two red-square locations is preceded by either a symmetrical or asymmetrical pattern. This design ensures that participants are familiarized with both task components before the full assessment begins. As in the OSPAN task, timing constraints are individually tailored based on performance during the symmetry-judgment practice to discourage strategic rehearsal.

### 4.3 Procedure

As we explained in the background section, the study consisted of one online and two laboratory sessions. In the first session (the online one), participants completed only the two WMC tasks: OSPAN and SymSPAN. Each of the WMC tasks was preceded by a practice task.

Similar to previous studies [e.g. 6,8], after completing the WMC tasks, participants were asked to give their subjective assessment of their performance and the conditions while doing the task. For the first assessment, they were asked if the conditions enabled them to complete the tasks to their best abilities, instructing them to choose one of the three options (conditions could be described as follows: interfering with task performance, therefore task performance was below abilities; neutral for task performance, or favourable). Only participants who reported optimal conditions (suggesting reliable and valid task performance) were considered for the laboratory sessions; in total, 6 participants did not meet this criterion. After the online session, groups of participants with high, low, and medium WMC were randomly selected from the online sample and invited to participate in the laboratory sessions.

The OSPAN and SymSPAN tasks had the same form as in the online session. In both laboratory sessions, which were conducted one week apart, the WMC tasks were administered after the vigilance task, which measured the frequency of IAMs and IFTs. During the vigilance task, participants were asked to perform an N-back task. In the low cognitive demands condition, it was a 1-back task, whereas it was a 3-back task in the high cognitive demands condition. The administration of the 1-back versus 3-back task was counterbalanced across the two laboratory sessions. If a participant undertook the 1-back task during the first laboratory session, they always undertook the 3-back task during the second laboratory session, and vice versa.

**Fig. 1:**
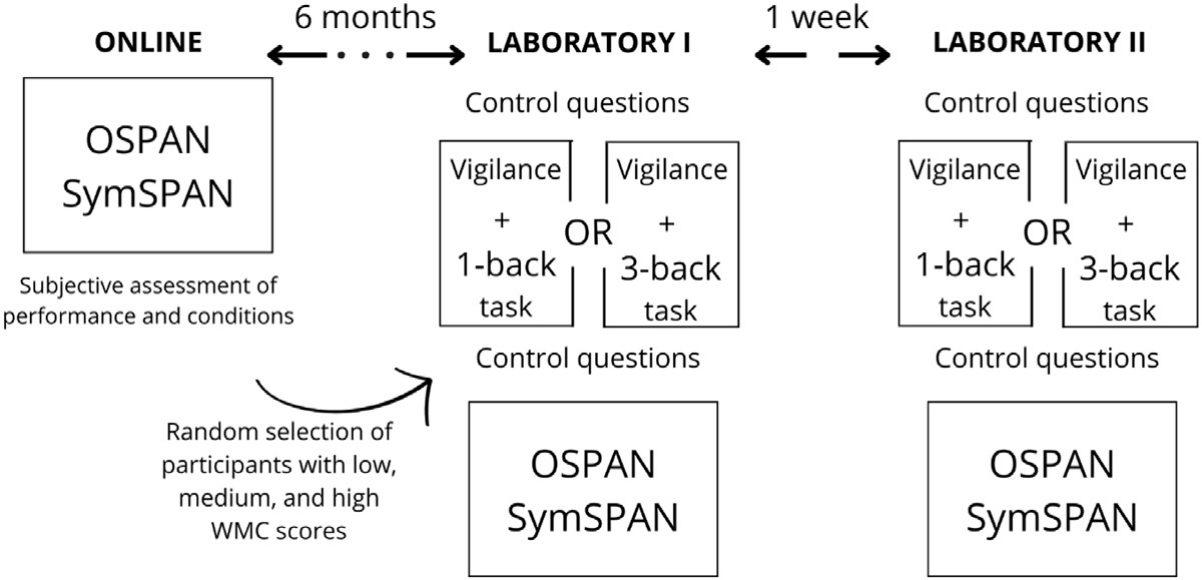
Working memory capacity measures in the context of other tasks. Online and laboratory sessions outline.

Before and after the vigilance task, participants received control questions regarding their mood and their levels of mental and physical fatigue. The outline of the procedure is presented in Fig. 1.

## Limitations

While the presented dataset offers valuable opportunities for research on working memory across different testing environments, some limitations should be acknowledged. First, the sample predominantly comprised younger female participants, which restricts generalizability of the findings to broader populations. Additionally, groups such as older adults, economically disadvantaged individuals, or those with lower education levels may be underrepresented as we did not systematically control for socioeconomic status or educational backgrounds. These demographic variables may potentially influence cognitive task performance, and future research should aim for greater demographic balance.

Second, the dataset did not control for clinical conditions such as depression [14,15] or ADHD [16,17], which can contribute to variability in task performance.

Third, technical limitations associated with the Inquisit software—particularly potential variability in response latency due to differences in participants’ devices and system configurations—may introduce measurement variability, which could affect the precision of response time data. However, previous research has shown that Inquisit provides response time data comparable to those obtained in laboratory settings [18]. Moreover, prior work by Hicks et al. [19] has already demonstrated the feasibility of administering the OSPAN online, supporting the validity of such remote assessments.

Finally, the n-back task was administered prior to the complex span tasks in the laboratory. This sequencing could lead to fatigue, practice effects, or differences in perceived task difficulty across participants, potentially confounding direct comparisons. Moreover, the n-back task was only performed in the laboratory setting, which limits the possibility of comparing its performance across online and offline environments. Recognizing these limitations may be important for appropriately contextualizing the findings and guiding researchers in implementing methodological adjustments that account for contextual effects.

## Ethics Statement

The University Research Ethics Committee approved the study (no: KE/39_2021). Written consent for participation was obtained prior to data collection. Participants were informed that they were free to withdraw from the study at any point.

## CRediT Author Statement

**Michał Wereszczyński:** Conceptualization, Data curation, Methodology, Resources, Software, Validation, Writing—original draft, Writing—review and editing. **Paulina Chwiłka:** Investigation (online & laboratory), Project administration, Data curation, Resources, Visualization, Writing—original draft, Writing review and editing. **Ewa Ilczuk:** Conceptualization, Investigation (online), Methodology, Project administration, Resources, Visualization, Writing—original draft, Writing—review and editing. **Ewa Smołka:** Writing—review and editing, Writing—original draft, Visualization. **Sezin Öner:** Visualization, Writing—original draft, Writing—review and editing. **Krystian Barzykowski:** Conceptualization, Funding acquisition, Investigation (online), Methodology, Project administration, Data curation, Resources, Supervision, Writing—original draft, Writing—review and editing;

## Data Availability

RODBUKWorking memory (capacity) behavioral tasks in online and laboratory settings: data from SymSPAN and OSPAN tasks (Original data).
